# *BRAF*非V600E突变型肺癌靶向治疗的策略探索

**DOI:** 10.3779/j.issn.1009-3419.2021.101.51

**Published:** 2022-02-20

**Authors:** 红霞 张, 金生 高, 伟 郭, 博 俞, 海涛 杨, 雨桃 刘

**Affiliations:** 1 252000 聊城，聊城市人民医院全科医学科 Department of General Practice, Liaocheng People's Hospital, Liaocheng 252000, China; 2 637000 南充，仪陇县人民医院肿瘤科 Department of Medical Oncology, Yilong People's Hospital, Nanchong 637000, China; 3 252000 聊城，聊城市第四人民医院家庭病房科 Family ward Department, Liaocheng Fourth People's Hospital, Liaocheng 252000, China; 4 252000 聊城，聊城市人民医院呼吸与危重症医学科 The Department of Pulmonary and Critical Care Medicine, Liaocheng People's Hospital, Liaocheng 252000, China; 5 100021 北京，国家癌症中心/国家肿瘤临床医学研究中心/中国医学科学院北京协和医学院肿瘤医院内科/抗肿瘤分子靶向药物临床研究北京市重点实验室 National Cancer Center/National Clinical Research Center for Cancer/Cancer Hospital, Chinese Academy of Medical Sciences Peking Union Medical College, Beijing Key Laboratory of Clinical Study on Anticancer Molecular Targeted Drugs, Beijing 100021, China

**Keywords:** *BRAF*非V600E突变, 肺肿瘤, 靶向治疗, *BRAF* non-V600E mutant, Lung neoplasms, Targeted therapy

## Abstract

**背景与目的:**

达拉非尼+曲美替尼/达拉非尼靶向治疗已被批准用于V-RAF小鼠肉瘤病毒癌基因同源B1（V-RAF murine sarcoma viral oncogene homolog B1, *BRAF*）发生第600位密码子上缬氨酸的氨基酸替代（amino acid substitution for valine at position 600, V600E）的肺癌患者，针对携带*BRAF*非V600E突变的肺癌患者的靶向治疗策略尚未确定。本研究拟探讨*BRAF*非V600E突变型肺癌靶向治疗的疗效，为临床治疗提供参考。

**方法:**

计算机检索PubMed、Cochrane Library、Embase、Web of Science、Clinicaltrials.gov、CBM、CNKI、万方数据库。收集*BRAF*非V600E突变型肺癌靶向治疗相关文献，对纳入文献进行描述性分析。

**结果:**

符合纳入标准的文献10篇，包括3篇队列研究和7篇个案报道。18例*BRAF*非V600E突变型肺癌患者对维莫非尼无效；1例应用维莫拉非尼后获得部分缓解（partial response, PR），5例患者对BRAF抑制剂无反应；9例患者在曲美替尼单药治疗后潜在临床获益率为34%；7例患者对达拉非尼联合曲美替尼在无进展生存期（progression-free survival, PFS）上均有不同程度的获益；1例患者对索拉非尼有效。

**结论:**

目前*BRAF*非V600E突变靶向治疗仍无标准治疗规范，挑战在于*BRAF*基因的异质性突变，不同的突变类型对靶向治疗的反应不同，真实世界研究证据匮乏，有必要开展进一步的大样本高质量研究为临床治疗方案的选择提供参考。

## Abstract (en)

**Background and objective:**

Dabrafenib+Trametinib/Dabrafenib targeted therapy has been approved for V-RAF murine sarcoma viral oncogene homolog B1 with amino acid substitution for valine at position 600 (*BRAF* V600E) in lung cancer patients, however, the targeted therapy strategy for lung cancer patients with *BRAF* non-V600E mutations has not been determined yet. This study intends to explore the efficacy of targeted therapy for *BRAF* non-V600E mutant lung cancer, and provide a reference for clinical treatment.

**Methods:**

Computer search of PubMed, Cochrane Library, Embase, Web of Science, Clinicaltrials.gov, CBM, CNKI, Wanfang database. Collect the relevant literature relevant on the targeted therapy of *BRAF* non-V600E mutant lung cancer, and conduct a descriptive analysis of the included literature.

**Results:**

There were 10 articles that met the inclusion criteria, including 3 cohort studies and 7 case reports. 18 patients with *BRAF* non-V600E mutant lung cancer were ineffective to vermurafenib; 1 patient obtained partial response (PR) after applying vermurafenib, 5 patients did not respond to BRAF inhibitors; 9 patients showed a potential clinical benefit rate of 34% after monotherapy with trametinib; 7 patients have different degrees of benefit from dabrafenib and trametinib on progression-free survival (PFS); 1 patient is effective to sorafenib.

**Conclusion:**

At present, there is no standard treatment specification for *BRAF* non-V600E mutation targeted therapy. The challenge lies in the heterogeneous mutation of *BRAF* gene. Different mutation types respond differently to targeted therapy. In addtion, real-world research evidence is scarce, so it is necessary to carry out further large-sample high-quality research to provide reference for clinical practice.

V-RAF小鼠肉瘤病毒癌基因同源B1（V-RAF murine sarcoma viral oncogene homolog B1, *BRAF*）突变在肺癌中是一种罕见突变，主要发生在组织学类型为腺癌的患者中，在非小细胞肺癌（non-small cell lung cancer, NSCLC）中的患病率在1.5%-3.5%之间。*BRAF*基因编码丝氨酸/苏氨酸激酶，属于调节细胞生长的大鼠肉瘤-快速加速纤维肉瘤-丝裂原活化蛋白激酶-细胞外信号调节激酶（rat sarcoma-rapidly accelerated fibrosarcoma-mitogen activated protein kinase kinase -extracellular signal regulated kinase, RAS-RAF-MEK-ERK）信号通路^[1]^。*BRAF*中最常见的突变是第600位密码子上的缬氨酸到谷氨酸氨基酸的替代（amino acid substitution for valine at position 600, V600E），代表着一种驱动突变，有效地针对选择性BRAF和（或）MEK抑制剂^[2]^。在肺癌中，V600位点以外的突变至少占*BRAF*突变的一半^[3]^。然而，到目前为止，精准医学的努力主要集中在V600亚组上，目前各指南中靶向治疗只被批准用于V600E突变型肺癌患者，针对携带*BRAF*非V600E突变的肺癌患者的靶向治疗策略尚未确定。

具有*BRAF*非V600E突变的肺癌人群的靶向治疗方面的文献有限且零散，故笔者在本研究通过检索文献的方式收集整理*BRAF*非V600E突变的肺癌靶向治疗的相关研究。预检索显示有关*BRAF*非V600E突变的文献较少，研究间存在临床异质性和方法学异质性，不具备合并进行*Meta*分析的条件，故笔者在本文仅对具有*BRAF*非V600E突变的肺癌患者的个案报道和临床研究中相关部分进行描述性分析。

## 1 资料与方法

### 1.1 纳入与排除标准

对纳入研究的病例、干预措施及研究类型的选择标准：病理诊断为肺癌（所有组织学亚型），分子基因检测为*BRAF*非V600E突变。干预措施：BRAF抑制剂或MEK抑制剂等靶向治疗。研究主要终点：无进展生存期（progression-free survival, PFS）。研究类型：随机对照研究、队列研究、个案报道、真实世界研究、非随机对照研究、病例对照研究等。排除标准：仅有研究计划、未报道研究结果的研究。

### 1.2 文献检索

根据纳入与排除标准，制定检索策略。系统检索PubMed、Cochrane Library、Embase、Web of Science、Clinicaltrials.gov、CBM、CNKI、万方数据库中涉及*BRAF*非V600E突变的肺癌靶向治疗的相关文献。检索语种为英语和汉语。以“Lung Neoplasm”、“Pulmonary Neoplasm”、“Lung Cancer”、“Pulmonary Cancer”、“BRAF non V600E”、“non-V600E BRAF”、“non BRAF V600E”为检索词，检索PubMed、Cochrane Library、Embase、Web of Science、Clinicaltrials.gov等英文数据库；以“肺癌”“肺肿瘤”“BRAF非V600E”为关键词或题名检索CBM、CNKI、万方等中文数据库。同时我们对纳入研究的参考文献进行进一步评估以发现可能符合要求的研究。

### 1.3 数据的提取与分析

由两名评价者背对背提取数据，并交叉核对，如遇争议，由第三名评价者裁决。文献中个案报道提取信息包括年龄、性别、吸烟史、组织学类型、分期、*BRAF*突变类型、治疗方案、PFS。

### 1.4 方法

将符合纳入标准的各项研究用描述性分析方法进行表述。

## 2 结果

### 2.1 文献检索结果

初检共获取文献83篇，其中PubMed 12篇，Cochrane Library 6篇，Embase 30篇，Web of Science 5篇，Clinicaltrials.gov、CBM、CNKI及万方等中文数据库未检索到相关文献，检索纳入研究的参考文献列表获取30篇。查重、阅读题目及摘要和全文后，纳入符合纳入标准的文献10篇（图 1）。

**图 1:**
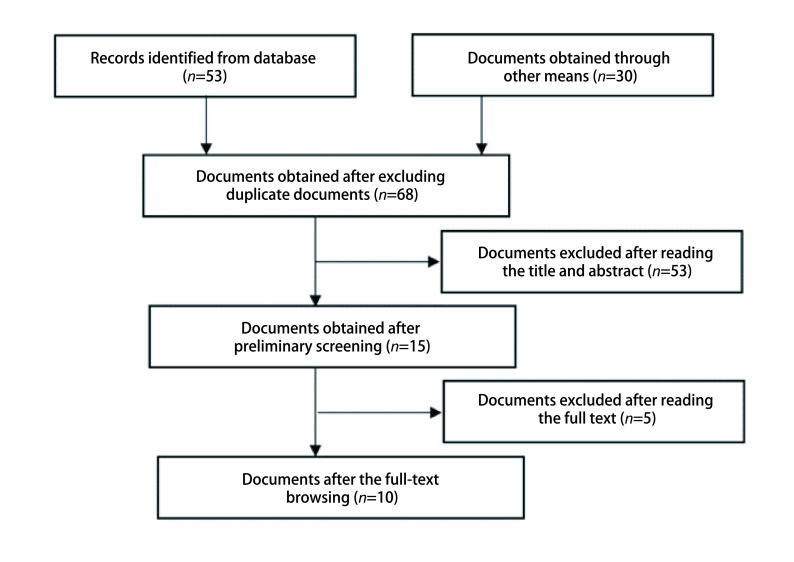
文献筛选流程图 Flow chart of literature screening

### 2.2 文献分析

纳入的10篇文献中，3篇是队列研究^[4-6]^，7篇是个案报道^[7-13]^。

1篇队列研究为法国国家癌症研究所启动的ACSé试验的NSCLC队列的最终结果，该研究共入选118例NSCLC患者，其中101例携带*BRAF* V600E，17例携带其他潜在激活突变（4例G466，4例G469，1例G596，5例K601，3例N581）。维莫非尼对*BRAF* V600E突变的NSCLC患者有较好的缓解率和延长的PFS，但对其他*BRAF*突变的NSCLC患者无效^[4]^。另1篇队列研究为欧洲EURAF队列，该队列纳入29例V600E突变及6例非V600E突变，包括G466V、G469A、G469L、G596V、V600K和K601E。除1例携带G596V的患者用维莫拉非尼获得PR外，其余患者对BRAF抑制剂无反应^[5]^。此外，Johnson等^[6]^分析了32例*BRAF*非V600E突变型肿瘤患者应用曲美替尼单药治疗的疗效，其中包括9例肺腺癌患者，主要研究终点为客观缓解率（objective response rate, ORR），研究结果观察到3%的无效缓解率和34%的潜在临床受益率。但是该人群大多数经过了多线治疗，并且有患者存在共突变因素，均可影响患者ORR。在所有肺癌患者中，他们还记录了1例*BRAF* G469A突变患者，该患者持续治疗22个周期（20.4个月），无进展。

7篇为个案报道，纳入9例患者，个案报道的病例提取信息见表 1。其中，1例*BRAF* G469L突变型的Ⅳ期肺腺癌患者，口服维莫非尼靶向治疗无效^[8]^，和ACSé试验^[4]^的结论一致。1例*BRAF* G469R突变的Ⅳ期肺腺癌病例，该患者经七线治疗，应用索拉菲尼后锁骨上肿物出现迅速而显著的坏死转变，肺部病灶稳定维持6个月^[12]^。其他7例患者均为Ⅳ期肺癌患者，其中6例组织学类型为腺癌，1例为肺大细胞神经内分泌癌。治疗方案选择为BRAF抑制剂（达拉非尼）±MEK抑制剂（曲美替尼），其中6例为联合用药，1例为曲美替尼单药治疗，在PFS上均有不同程度的获益，提示*BRAF*非V600E突变型肺癌患者可能对BRAF抑制剂联合MEK抑制剂敏感。

**表 1:** 个案报道中*BRAF*非V600突变患者基线临床特征 Baseline clinical characteristics of *BRAF* non-V600 mutation patients in case reports

Age	Sex	Smoking history	Histological type	Stage	*BRAF* mutation type	Treatment	PFS
48	Male	No	Adenocarcinoma	Ⅳ	G469V	Dabrafenib+trametinib	2 mon
63	Female	Yes	Adenocarcinoma	Ⅳ	L597R	Dabrafenib+trametinib	> 12 mon
60	Male	Yes	Adenocarcinoma	Ⅳ	D594G	Trametinib	4 mon
62	Female	Yes	Adenocarcinoma	Ⅳ	G469A	Dabrafenib+trametinib	6 mon
76	Male	Yes	Adenocarcinoma	Ⅳ	G469L	Vemurafenib	14 d
56	Female	Yes	Adenocarcinoma	Ⅳ	G469R	Sorafenib	6 mon
75	Female	No	Adenocarcinoma	Ⅳ	T599D	Dabrafenib+trametinib	> 4 mon
69	Male	Yes	Adenocarcinoma	Ⅳ	G469A, T604C	Dabrafenib+trametinib	15 mon
69	Male	Yes	Large cell neuroendocrine carcinoma	Ⅳ	G469R	Dabrafenib+trametinib	> 15 mon
BRAF: V-RAF murine sarcoma viral oncogene homolog B1; PFS: progression-free survival.							

## 3 讨论

BRAF是RAF丝氨酸/苏氨酸激酶家族的成员，在RAS-RAF-MEK-ERK信号通路中位于RAS下游：被RAS激活后，BRAF磷酸化双特异性的MEK，导致ERK的激活，最终导致ERK信号通路的激活，这是调节细胞生长的关键通路^[1]^。根据信号机制和激酶活性将BRAF突变分为三类：V600E突变的激酶激活单体（Ⅰ类）、激酶激活二聚体（Ⅱ类）和激酶失活异二聚体（Ⅲ类）。BRAF抑制剂和MEK抑制剂的联合治疗目前被用于有效阻断具有一类突变的丝裂原活化蛋白激酶（mitogen-activated protein kinase, MAPK）通路，联合治疗可能对BRAF非V600E突变也有活性^[14]^。国家癌症综合网络、欧洲医学肿瘤学学会及中国临床肿瘤学会指南认可对NSCLC进行*BRAF*基因检测。这些指南建议对携带*BRAF* V600E突变的患者在一线或后续治疗中使用达拉非尼和曲美替尼，因为它们在NSCLC一线和后续治疗中表现出了疗效^[15, 16]^。但是NSCLC中约50%的*BRAF*突变是非V600E突变型的，而且这些不同的突变对BRAF±MEK抑制剂的反应性不同^[3, 17]^。本文纳入的病例也体现了上述观点。所以，针对*BRAF*非V600E突变靶向治疗的挑战在于*BRAF*基因的异质性突变，一方面不同的*BRAF*非V600E突变类型具有不同的生物学功能；另一方面*BRAF*非V600E突变可能还有许多意义未知的突变体。因此，需要做更多的研究来更好地理解不同的变异。这样看来，*BRAF*突变的多样性和功能异质性，阻碍了*BRAF*非V600E突变型肺癌治疗策略的发展。另外，在欧洲EURAF队列6例BRAF非V600E患者中，1例G596V患者在维莫非尼治疗后出现部分缓解（partial response, PR）^[5]^，而本文纳入的G469L突变患者在服用维莫非尼后病情出现迅速进展，似乎提示非V600E突变位于BRAF激酶结构域激活片段之外的肿瘤对BRAF抑制剂是无效的，或许支持位于激活环（密码子596-600）内突变的患者对BRAF抑制剂敏感。但是INCA的ACSé试验中17例BRAF非V600E突变患者均对维莫非尼无效，其中包括1例G596突变患者，无法完全支持上述观点，有必要在未来的临床试验中进行更多的大样本的研究加以验证。

*BRAF*突变在肺癌中的预后意义仍不确定，值得注意的是，关于这个问题，现有的研究探索产生了不一致的结果，一些研究表明非V600E组的PFS及总生存期（overall survival, OS）更长，而另一些研究的结果正好相反。一项法国的多中心回顾性研究发现V600E突变是一个不利的预后因素，在多变量分析中与较短的OS显著相关（HR=2.18, *P*=0.014）；特别是具有*BRAF* V600E突变的患者比非V600E突变的患者具有更短的中位PFS和OS（分别为15.2个月与52.1个月，*P*=0.001；分别为29.3个月与72.4个月，*P*=0.001）^[18]^。Cardarella等^[19]^研究者也得到了类似的结论。而另一项回顾性研究的结论却截然相反，该研究纳入63例*BRAF*突变型肺腺癌患者，36例患者有*BRAF* V600E突变，27例患者有非V600E突变。V600E突变患者和非V600E突变患者在年龄、性别或初诊阶段方面差异无统计学意义。与携带非V600E突变的肿瘤患者相比，携带V600E突变的肿瘤患者更有可能是轻度/从不吸烟的患者（*P*=0.007）。共检测到5种*BRAF*基因突变类型，分别为：V600E（57%）、G469A（22%）、D469V（13%）、D594G（6%）和V600M（2%），其中V600E突变肺癌患者在早期肺癌切除后3年的OS与非V600E突变肺癌患者相似（67% *vs* 75%, *P*=0.42）。在IIIb期或Ⅳ期*BRAF*突变肺腺癌患者中，V600E突变的患者比非V600E突变的患者有更长的3年OS（24% *vs* 0%, *P* < 0.001）^[20]^。类似的研究比如欧洲EURAF队列，该队列纳入29例（83%）V600E突变，6例（17%）非V600E突变，包括G466V、G469A、G469L、G596V、V600K和K601E。对于包括化疗在内的一线治疗，V600E组的PFS为37周（9.3个月），非V600E组为6周（1.5个月）。V600E组OS为101周（25.3个月），非V600E组为47周（11.8个月）。提示非V600E患者的PFS及OS比V600E患者短^[5]^。而一项来自中国的研究根据*BRAF*突变类型分析了105例接受一线化疗方案治疗的IIIb-Ⅳ期患者的生存结果。其中51例、32例和21例分别存在1类、2类和3类*BRAF*突变。*Kaplan-Meier*和*Log-rank*分析显示，三种*BRAF*突变类型之间的总体存活率相当，1类、2类和3类突变的中位总生存时间分别为28.6个月、13.9个月和20.2个月（*P*=0.585）^[21]^。一项来自德国癌症中心的研究^[22]^也获得了类似的结果，他们的研究纳入了72例*BRAF*突变的肺癌患者，*BRAF*突变亚型显示31例存在p.V600E突变，41例存在18种不同功能Ⅱ类/Ⅲ类*BRAF*突变亚型，研究结果显示*BRAF*突变的三个功能类别之间的生存终点差异无统计学意义。对于这些研究结论的不同，可能是受到不太理想的研究设计的影响，特别是患者数量较少以及未能区分不同的治疗方案。

虽然一些研究表明BRAF/MEK抑制剂对*BRAF*非V600E突变型肺癌治疗有效，但考虑到这两种突变的差异，对于非V600E突变的肺癌患者可能会有更好的靶向免疫治疗，需要做更多的研究来验证。近些年免疫检查点抑制剂（immune-checkpoint inhibitor, ICI）在肺癌的各线治疗中取得了巨大的成果，成为治疗*BRAF*非V600E突变的潜在新疗法。一项基于真实世界的研究^[23]^针对ICI对携带BRAF、人类表皮生长因子受体-2（human epidermal growth factor receptor-2, *HER-2*）、间质-上皮细胞转化因子（mesenchymal-epithelial transition factor, *MET*）和转染原癌基因（transfection proto-oncogene gene, *RET*）等突变基因的NSCLC的疗效做了回顾性研究，结论提示*BRAF*非V600E突变型NSCLC（*n*=18）的有效率为35%。Offin等^[7]^对*BRAF*非V600E突变型肺癌的分子特征、免疫表型和对ICI的反应进行分析，36例*BRAF*非V600E突变的患者接受ICI治疗[Nivolumab (*n*=25), Pembrolizumab (*n*=5), Atezolizumab (*n*=2), Ipilimumab/nivolumab (*n*=4)]，ORR为22%（8/36）。接受ICI治疗的非V600E和V600E患者的ORR差异无统计学意义（*P*=0.66）。*BRAF*非V600E患者的OS为1.7年，而V600E患者为2.5年（HR=1.25, *P*=0.38）。接受ICI治疗的BRAF非V600E肺癌患者的OS（2.4年）高于未接受ICI的患者（1.2年；HR=0.60，*P*=0.04）。与*BRAF* V600E相比，*BRAF*非V600E突变型肺癌对晚期ICI有较高的应答率和较好的OS。Rittberg^[24]^报道了1例*BRAF* G469A突变的IIIb期肺腺癌患者，该患者为61岁女性，程序性死亡配体-l（programmed death ligand-1, PD-L1） > 50%，伴间变淋巴瘤激酶基因（anaplastic lymphoma kinase, *ALK*）易位、表皮生长因子受体（epidermal growth factor receptor, *EGFR*）突变。一线治疗为根治性放疗（66 Gy/33 f）联合同步化疗（顺铂和依托泊苷），治疗后CT显示右肺门肿块改善，但双肾上腺转移瘤进展，二线进行纳武利尤单抗治疗，PFS超过4年。因此，对这一癌基因亚群进行进一步的免疫治疗研究是有必要的。

本研究的局限性主要在于原始研究缺乏。目前无专门针对*BRAF*非V600E突变型肺癌靶向治疗的大样本高质量临床研究。现有证据主要从已发表的临床研究中进行亚组分析及个案报道的总结。真实世界研究尤其匮乏，部分真实世界的研究仅发表了摘要，研究信息不完整。

总体而言，目前*BRAF*非V600E突变型肺癌靶向治疗的证据较匮乏，尤其缺乏真实世界研究证据，而真实世界研究对于制定*BRAF*非V600E突变型肺癌患者的治疗策略尤为重要，有必要开展进一步的大样本高质量研究为临床治疗方案的选择提供参考。
